# Supplementary material for: Anti-Proliferative and Cytoprotective Activity of Aryl Carbamate and Aryl Urea Derivatives with Alkyl Groups and Chlorine as Substituents
Source: Molecules. 2022 Jun 4;27(11):3616. doi: 10.3390/molecules27113616 (PMC9182529; doi:10.3390/molecules27113616)
Supplement: Supplementary file 1 [file molecules-27-03616-s001.zip › molecules-1714360-supplementary.pdf]

Supplement

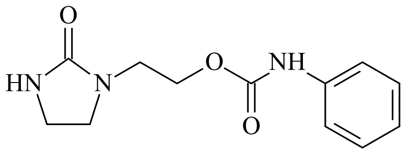

Compound IIIa
1H NMR (DMSO-d6)

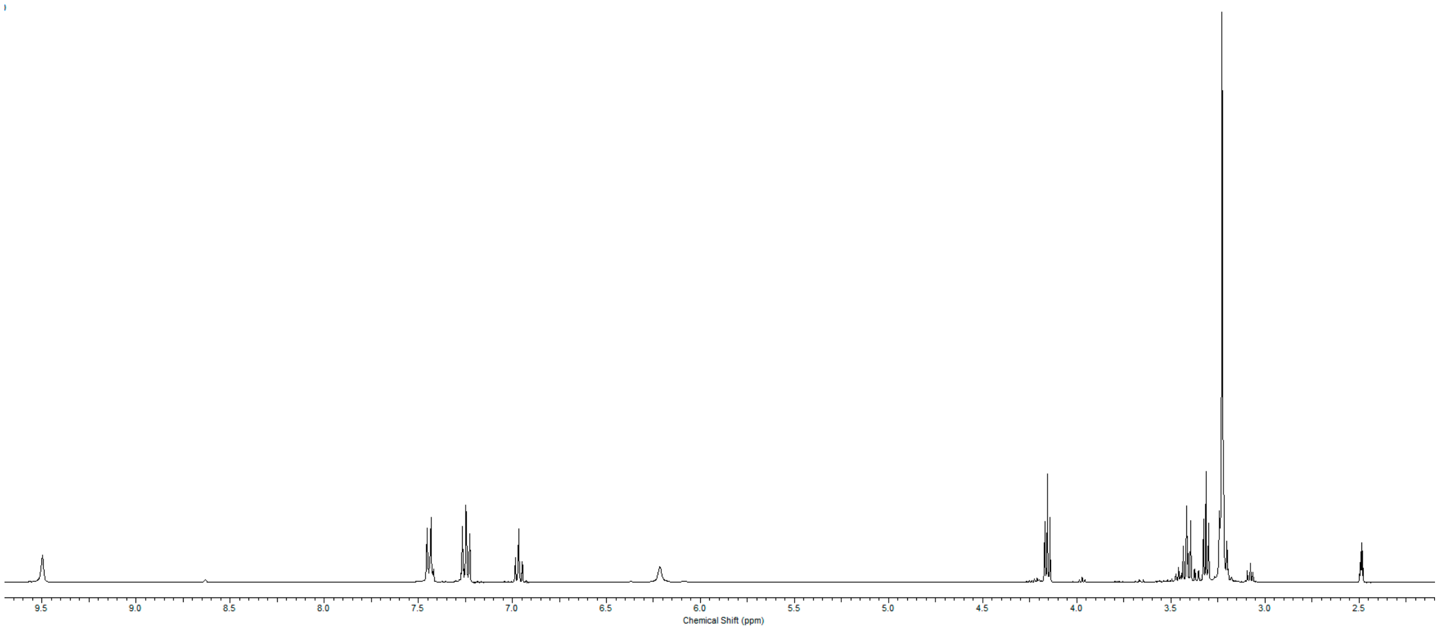

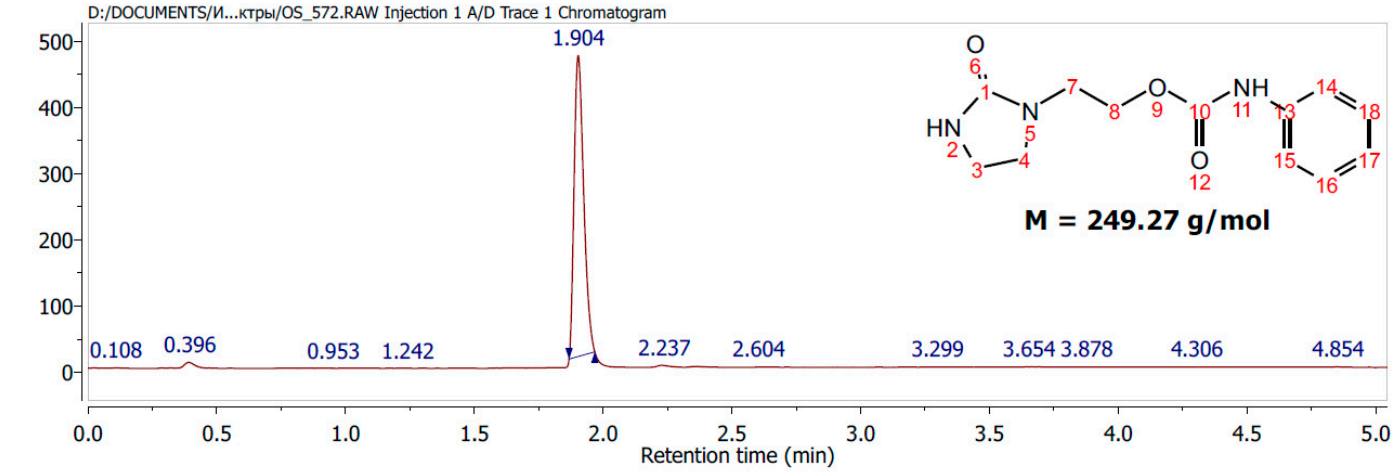

D:/DOCUMENTS/И...ктры/OS_572.RAW Injection 1 A/D Trace 1 Chromatogram
1.904
0.108
0.396
0.953
1.242
2.237
2.604
3.299
3.654 3.878
4.306
4.854
Retention time (min)
M = 249.27 g/mol

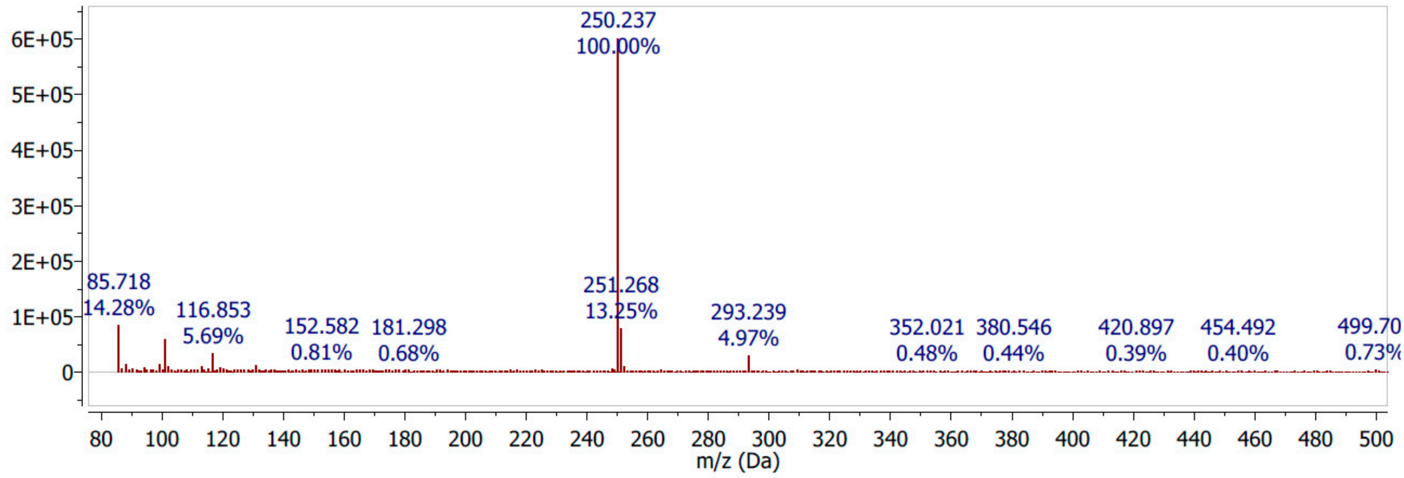

250.237
100.00%
85.718
14.28%
116.853
5.69%
152.582
0.81%
181.298
0.68%
251.268
13.25%
293.239
4.97%
352.021
0.48%
380.546
0.44%
420.897
0.39%
454.492
0.40%
499.70
0.73%
m/z (Da)

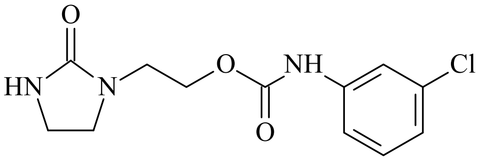

Compound IIIb
1H NMR (DMSO-d6)

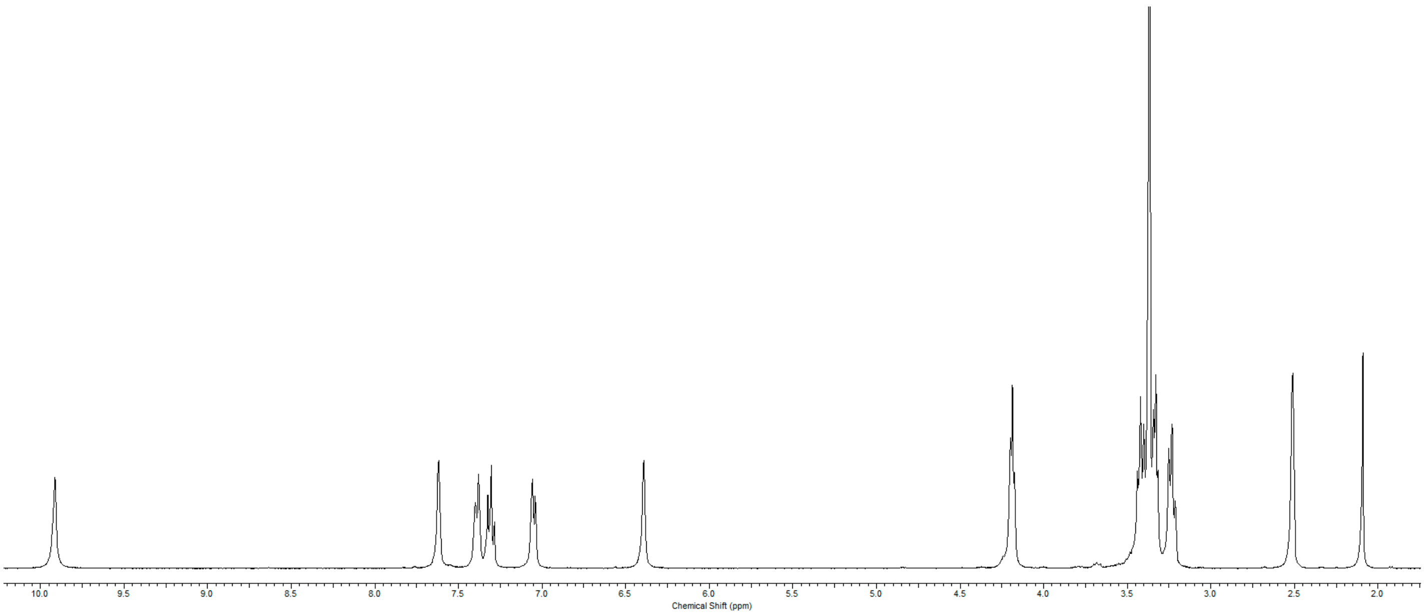

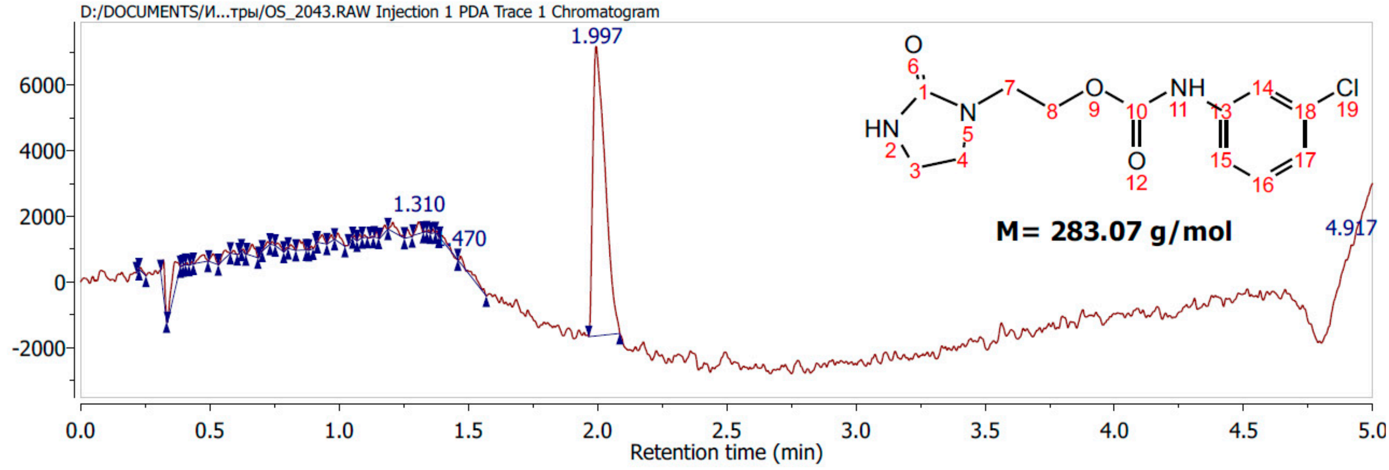
D:/DOCUMENTS/И...тры/OS_2043.RAW Injection 1 PDA Trace 1 Chromatogram
1.997
1.310
470
4.917
M= 283.07 g/mol
Retention time (min)

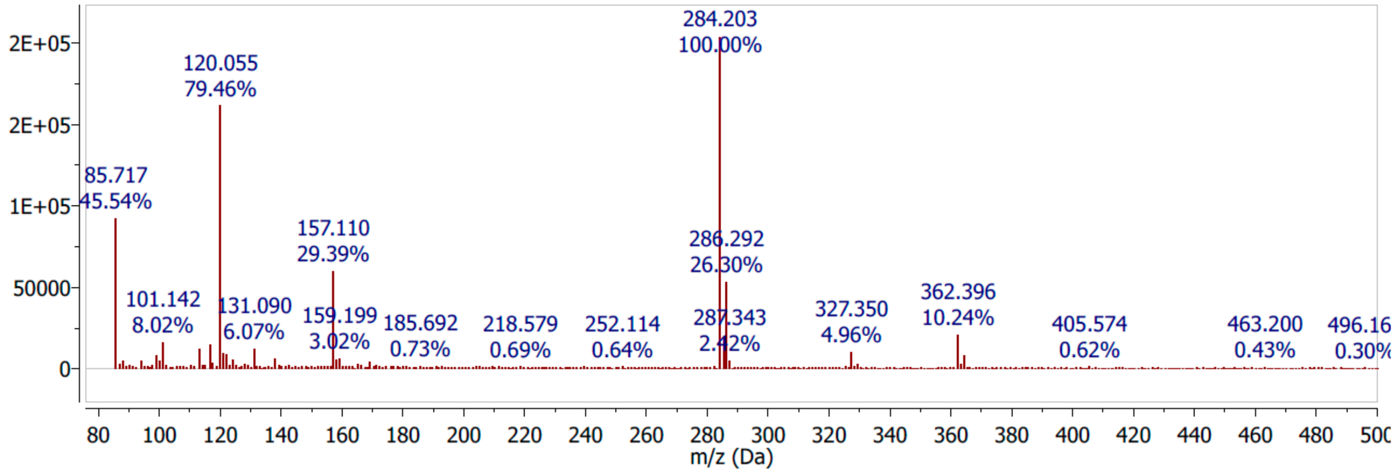
284.203
100.00%
120.055
79.46%
85.717
45.54%
157.110
29.39%
286.292
26.30%
101.142
8.02%
131.090
6.07%
159.199
3.02%
185.692
0.73%
218.579
0.69%
252.114
0.64%
287.343
2.42%
327.350
4.96%
362.396
10.24%
405.574
0.62%
463.200
0.43%
496.16
0.30%
m/z (Da)

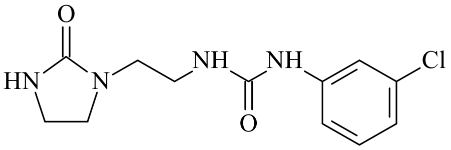

Compound IIIf
1H NMR (DMSO-d6)

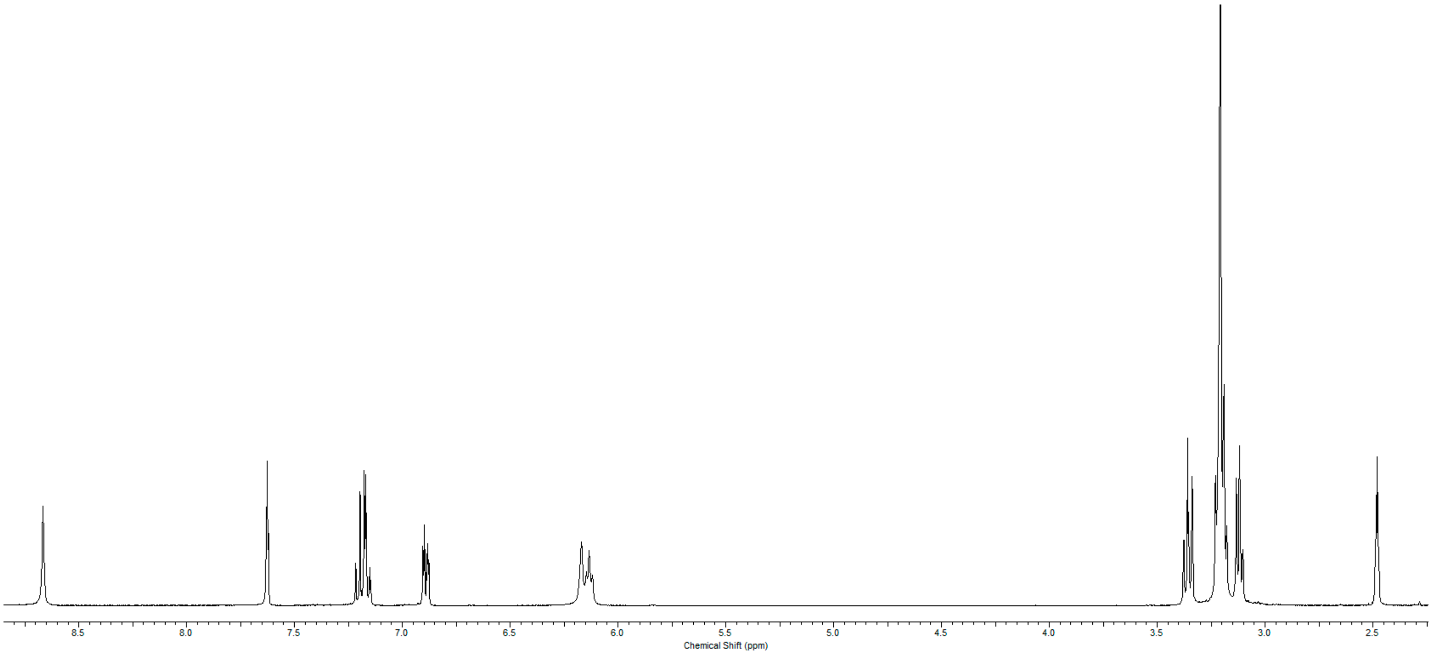

\

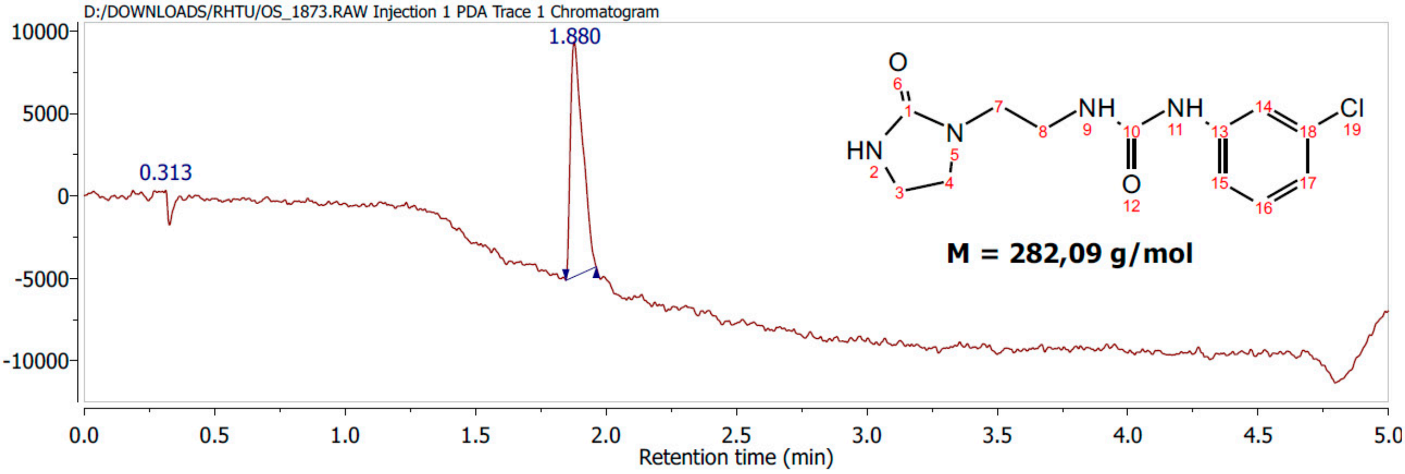

D:/DOWNLOADS/RHTU/OS_1873.RAW Injection 1 PDA Trace 1 Chromatogram
1.880
0.313
M = 282,09 g/mol
Retention time (min)

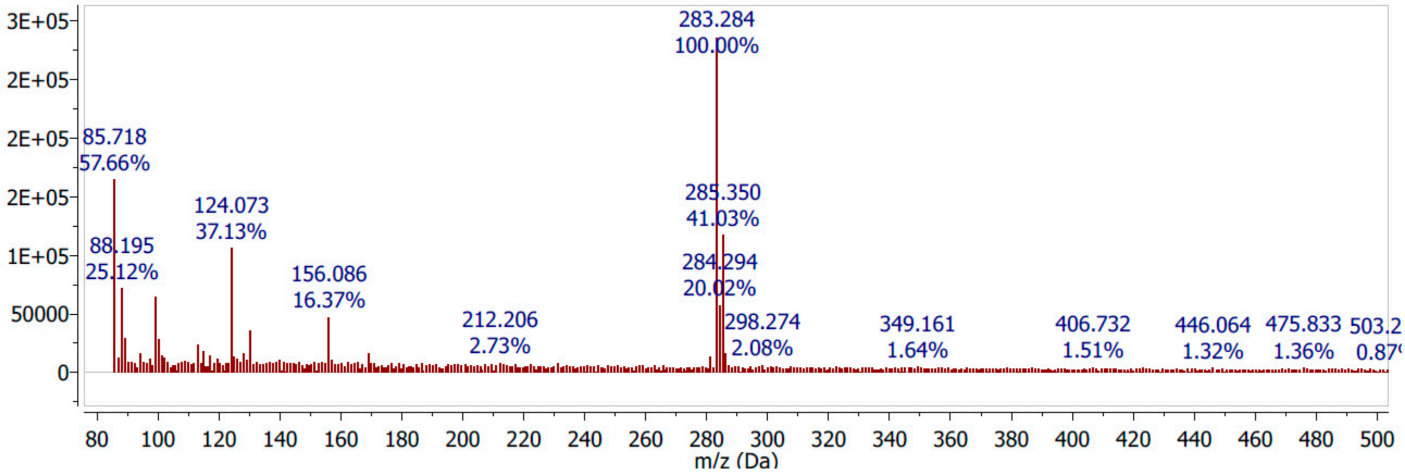

283.284
100.00%
85.718
57.66%
285.350
41.03%
124.073
37.13%
88.195
25.12%
156.086
16.37%
284.294
20.02%
212.206
2.73%
298.274
2.08%
349.161
1.64%
406.732
1.51%
446.064
1.32%
475.833
1.36%
m/z (Da)

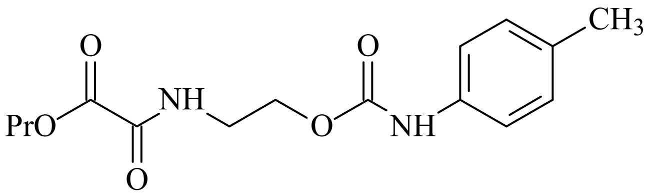

Compound IIb

1H NMR (DMSO-d6)

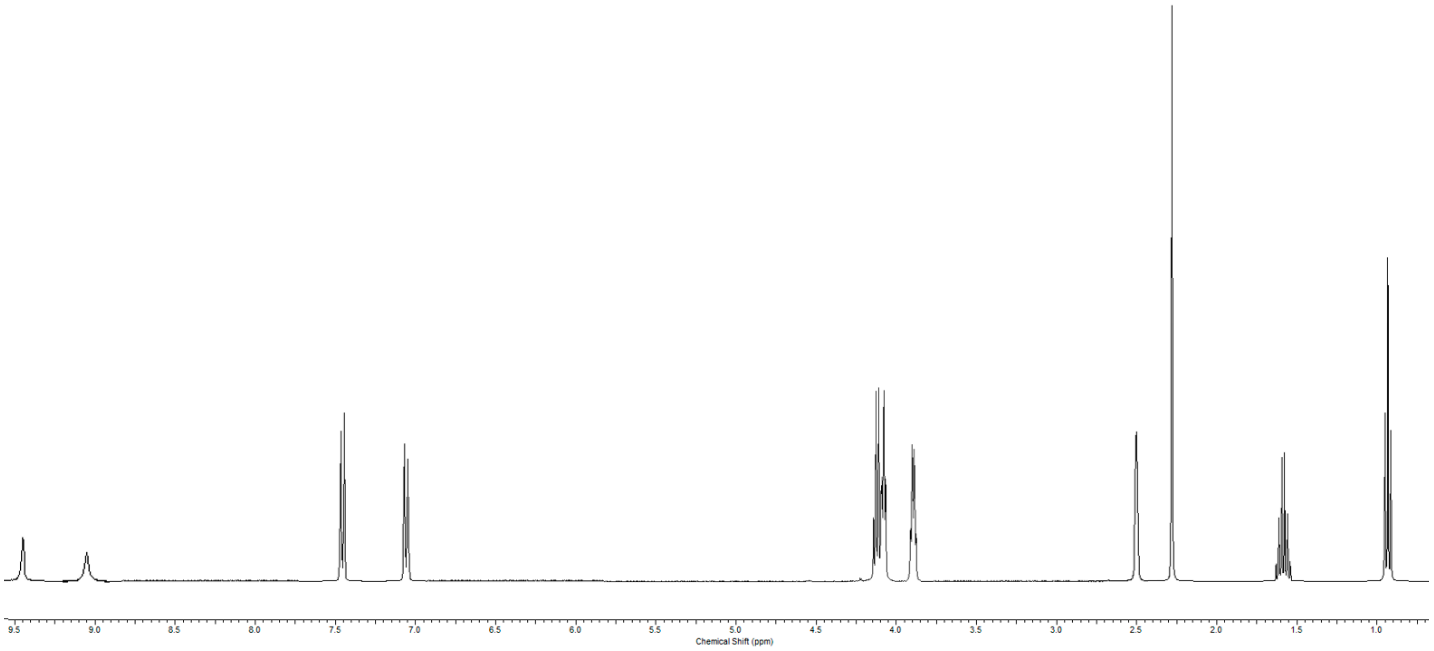

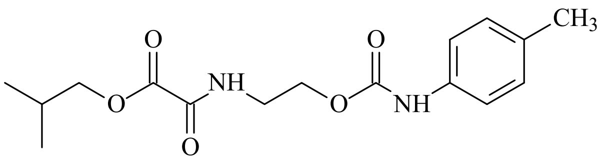

Compound IId
1H NMR (DMSO-d6)

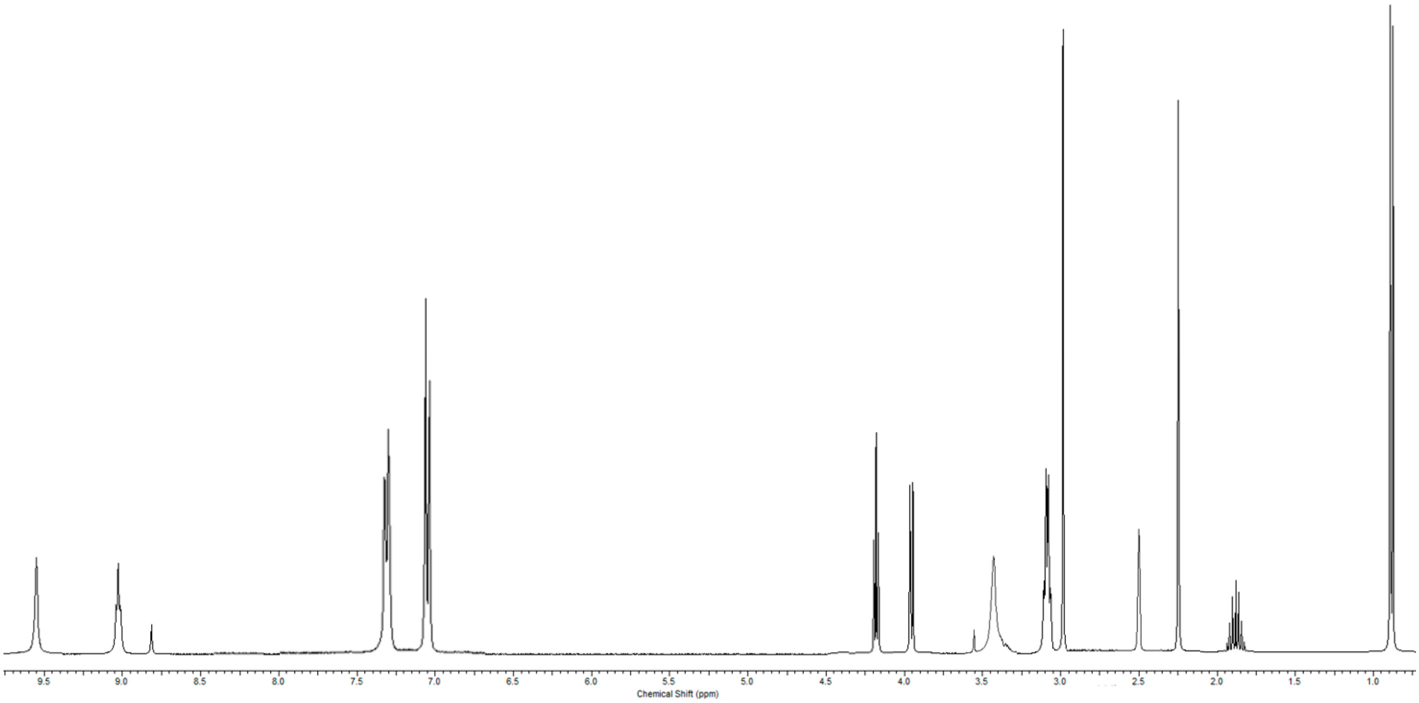

Figure S1. NMR spectroscopy data for the synthesized compounds.

Table S1. Affinities of the clusters for the A2AR receptor variant 5mzj

Cluster	Substance	Energy	Frequency
0	adenosine	-6.20±0.12	0.05
0	caffeine	-4.62±0.13	0.22
0	IIa	-4.85±0.07	0.02
0	IIIe	-4.55±0.49	0.02
1	caffeine	-4.71±0.15	0.13
1	IIb	-5.53±0.40	0.03
1	IIc	-5.65±0.21	0.02
1	IId	-4.90±nan	0.01
1	IIIf	-5.40±nan	0.01
1	IIIg	-5.30±nan	0.01
2	adenosine	-5.66±0.47	0.09
2	IIa	-4.60±nan	0.01
2	IIb	-4.40±0.26	0.04
2	IIc	-4.55±0.35	0.02
2	IIIa	-4.20±0.17	0.03
2	IIIc	-4.40±0.47	0.05

2	IIId	-4.87±0.37	0.07
2	IIIf	-4.40±0.34	0.05
2	IIIg	-4.65±0.64	0.02
2	IIIh	-4.69±0.32	0.12
3	adenosine	-4.94±0.05	0.05
3	IIa	-4.00±0.24	0.04
3	IIb	-4.23±0.36	0.04
3	IIc	-3.70±0.14	0.02
3	IIIa	-4.15±0.35	0.02
3	IIIb	-4.00±0.25	0.05
3	IIIc	-4.03±0.45	0.03
3	IIId	-4.26±0.62	0.06
3	IIIe	-3.74±0.27	0.09
3	IIIf	-3.92±0.13	0.06
3	IIIg	-4.00±0.42	0.05
3	IIIh	-3.96±0.46	0.06
**4**	adenosine	-5.37±0.42	0.25
4	caffeine	-4.53±0.09	0.22
4	IIa	-4.87±0.38	0.48
4	IIb	-4.80±0.38	0.26
4	IIc	-4.99±0.41	0.3
4	IId	-4.97±0.51	0.45
4	IIIa	-4.26±0.46	0.32
4	IIIb	-4.35±0.43	0.23
4	IIIc	-4.26±0.59	0.32
4	IIId	-4.43±0.48	0.27
4	IIIe	-4.25±0.50	0.25
4	IIIf	-4.58±0.46	0.29
4	IIIg	-4.28±0.42	0.24
4	IIIh	-4.58±0.60	0.26
5	adenosine	-5.30±0.19	0.12
5	caffeine	-4.60±0.09	0.06
5	IIa	-4.45±0.49	0.02
5	IIb	-4.56±0.48	0.16
5	IIc	-4.74±0.38	0.14
5	IId	-4.37±0.47	0.07
5	IIIa	-3.76±0.31	0.09
5	IIIb	-4.07±0.21	0.06
5	IIIc	-4.10±0.37	0.09
5	IIId	-4.35±0.36	0.1
5	IIIe	-4.17±0.31	0.08
5	IIIf	-4.47±0.42	0.05
5	IIIg	-4.04±0.21	0.09
5	IIIh	-4.43±0.40	0.05

6	adenosine	-5.56±0.39	0.1
6	IIa	-4.65±0.20	0.11
6	IIb	-4.98±0.16	0.07
6	IIc	-5.10±nan	0.01
6	IId	-4.73±0.50	0.07
6	IIIa	-4.47±0.40	0.13
6	IIIb	-4.58±0.36	0.15
6	IIIc	-4.22±0.16	0.06
6	IIId	-4.50±0.14	0.02
6	IIIe	-4.60±nan	0.01
6	IIIf	-4.90±0.26	0.04
6	IIIg	-4.65±0.24	0.05
6	IIIh	-5.10±0.37	0.05
7	adenosine	-4.85±0.21	0.02
7	IIa	-4.40±0.71	0.02
7	IIc	-4.26±0.15	0.05
7	IId	-3.90±0.42	0.02
7	IIIa	-3.60±0.46	0.03
7	IIIb	-3.92±0.29	0.04
7	IIIc	-3.85±0.13	0.05
7	IIId	-4.30±1.05	0.05
7	IIIe	-3.40±0.28	0.02
7	IIIf	-4.20±0.14	0.03
7	IIIg	-3.85±0.35	0.02
7	IIIh	-4.13±0.38	0.04
9	adenosine	-5.05±0.07	0.02
9	IIa	-4.42±0.23	0.05
9	IIb	-4.90±0.26	0.03
9	IIc	-4.87±0.29	0.1
9	IId	-5.35±0.07	0.02
9	IIIa	-3.90±0.44	0.08
9	IIIb	-4.23±0.12	0.06
9	IIIc	-3.85±0.07	0.02
9	IIId	-4.15±0.21	0.02
9	IIIe	-4.15±0.07	0.02
9	IIIf	-4.50±nan	0.01
9	IIIg	-3.97±0.23	0.03
9	IIIh	-4.40±0.20	0.05
10	adenosine	-5.50±0.00	0.02
10	caffeine	-4.73±0.11	0.21
10	IIa	-4.92±0.37	0.05
10	IIb	-5.02±0.22	0.04
10	IIc	-5.00±0.45	0.04
10	IId	-4.70±nan	0.01

10	IIIa	-4.40±0.44	0.03
10	IIIc	-4.50±nan	0.01
10	IIIe	-5.05±0.07	0.02
10	IIIf	-4.85±0.78	0.03
10	IIIg	-4.60±0.44	0.03
11	caffeine	-4.93±0.16	0.13
11	IId	-6.00±nan	0.01
11	IIIb	-5.25±0.64	0.02
11	IIIh	-5.10±nan	0.01
12	adenosine	-5.69±0.30	0.21
12	caffeine	-4.48±0.04	0.03
12	IIa	-4.57±0.42	0.03
12	IIb	-4.60±0.16	0.04
12	IIc	-4.60±0.16	0.05
12	IId	-4.31±0.48	0.09
12	IIIa	-4.33±0.37	0.07
12	IIIb	-4.42±0.32	0.12
12	IIIc	-4.20±0.29	0.16
12	IIId	-4.21±0.32	0.1
12	IIIe	-4.26±0.40	0.22
12	IIIf	-4.28±0.20	0.17
12	IIIg	-4.14±0.29	0.16
12	IIIh	-4.31±0.32	0.09
13	caffeine	-4.40±nan	0
13	IIa	-4.57±0.34	0.06
13	IIb	-4.35±0.50	0.07
13	IIc	-4.10±0.42	0.05
13	IId	-4.65±0.33	0.04
13	IIIa	-4.09±0.52	0.1
13	IIIb	-4.16±0.27	0.07
13	IIIc	-4.30±0.61	0.08
13	IIId	-4.19±0.45	0.17
13	IIIe	-4.26±0.53	0.08
13	IIIf	-4.33±0.38	0.08
13	IIIg	-4.42±0.38	0.06
13	IIIh	-4.78±0.58	0.06
14	adenosine	-4.95±0.21	0.02
14	IIa	-4.20±0.00	0.02
14	IIb	-4.10±0.00	0.03
14	IIc	-3.60±nan	0.01
14	IId	-4.04±0.34	0.07
14	IIIa	-4.07±0.47	0.03
14	IIIb	-4.12±0.04	0.05
14	IIIc	-3.67±0.15	0.03

14	IIId	-4.05±0.49	0.02
14	IIIe	-3.70±0.14	0.02
14	IIIf	-4.25±0.35	0.03
14	IIIg	-4.10±0.32	0.09
14	IIIh	-4.18±0.42	0.06
15	adenosine	-4.93±0.35	0.03
15	IIa	-3.90±0.38	0.04
15	IIb	-4.17±0.18	0.13
15	IIc	-4.20±0.34	0.11
15	IId	-4.08±0.32	0.09
15	IIIa	-3.92±0.34	0.04
15	IIIb	-4.01±0.32	0.13
15	IIIc	-3.78±0.37	0.06
15	IIId	-4.08±0.70	0.06
15	IIIe	-3.58±0.37	0.12
15	IIIf	-4.00±0.22	0.12
15	IIIg	-3.85±0.28	0.11
15	IIIh	-3.98±0.13	0.06

Table S2. Affinities of the clusters for the A2AR receptor variant 2ydo

Cluster	Substance	Energy	Frequency
0	adenosine	-5.44±0.42	0.03
0	caffeine	-4.55±0.13	0.17
0	IIa	-4.88±0.32	0.04
0	IIb	-4.40±nan	0.01
0	IId	-4.80±0.42	0.02
0	IIIb	-4.00±nan	0.01
0	IIIc	-4.50±nan	0.01
0	IIIe	-4.40±nan	0.01
2	adenosine	-5.28±0.37	0.04
2	caffeine	-4.92±0.27	0.19
2	IIb	-5.20±nan	0.01
2	IId	-6.25±0.64	0.02
2	IIId	-5.30±nan	0.01
2	IIIg	-5.00±nan	0.01
2	IIIh	-5.40±nan	0.01
3	adenosine	-5.13±0.42	0.2
3	caffeine	-4.37±0.06	0.18
3	IIa	-4.36±0.32	0.1
3	IIb	-4.23±0.36	0.09
3	IIc	-4.15±0.08	0.06
3	IId	-4.78±0.72	0.05
3	IIIa	-4.13±0.34	0.15
3	IIIb	-4.25±0.30	0.16

3	IIIc	-4.15±0.37	0.11
3	IIId	-4.26±0.26	0.09
3	IIIe	-4.04±0.27	0.15
3	IIIf	-4.37±0.36	0.08
3	IIIg	-4.17±0.36	0.15
3	IIIh	-4.56±0.34	0.12
4	adenosine	-4.00±nan	0.01
4	IIa	-3.70±nan	0.01
4	IIb	-4.33±0.49	0.03
4	IIc	-4.05±0.34	0.06
4	IId	-4.45±0.31	0.04
4	IIIa	-3.95±0.07	0.02
4	IIIb	-3.85±0.35	0.02
4	IIIc	-3.80±0.27	0.04
4	IIId	-4.00±0.10	0.03
4	IIIe	-3.85±0.35	0.02
4	IIIf	-4.25±0.28	0.09
4	IIIg	-4.00±0.28	0.02
4	IIIh	-4.27±0.25	0.03
5	adenosine	-4.65±0.49	0.01
5	caffeine	-4.30±0.00	0.02
5	IIa	-4.10±0.28	0.02
5	IIb	-4.38±0.22	0.03
5	IIc	-4.64±0.21	0.08
5	IId	-4.82±0.26	0.06
5	IIIa	-3.53±0.15	0.03
5	IIIb	-4.20±0.46	0.03
5	IIIc	-4.13±0.44	0.07
5	IIId	-4.12±0.21	0.09
5	IIIe	-3.70±nan	0.01
5	IIIf	-4.37±0.29	0.03
5	IIIg	-4.23±0.15	0.03
5	IIIh	-4.66±0.23	0.05
6	adenosine	-3.30±0.66	0.19
6	IIa	-4.40±0.44	0.04
6	IIb	-4.30±0.16	0.09
6	IIc	-4.24±0.22	0.12
6	IId	-4.39±0.29	0.08
6	IIIa	-3.76±0.32	0.05
6	IIIb	-4.01±0.32	0.08
6	IIIc	-3.58±0.21	0.06
6	IIId	-3.94±0.18	0.05
6	IIIe	-3.80±0.25	0.14
6	IIIf	-4.00±0.10	0.03

6	IIIg	-3.84±0.34	0.07
6	IIIh	-4.20±0.36	0.04
7	adenosine	-4.99±0.29	0.05
7	IIa	-4.63±0.15	0.06
7	IIb	-4.56±0.18	0.07
7	IIc	-4.95±0.35	0.02
7	IId	-4.80±0.29	0.05
7	IIIa	-4.22±0.26	0.04
7	IIIb	-4.17±0.13	0.04
7	IIIc	-4.05±0.19	0.04
7	IIId	-4.11±0.26	0.1
7	IIIe	-3.77±0.24	0.07
7	IIIf	-4.10±nan	0.01
7	IIIg	-4.15±0.07	0.02
7	IIIh	-4.20±0.35	0.03
8	adenosine	-4.42±0.87	0.14
8	IIa	-3.95±0.49	0.02
8	IIb	-4.53±0.47	0.03
8	IIc	-3.85±0.07	0.02
8	IId	-4.24±0.32	0.05
8	IIIa	-3.95±0.31	0.04
8	IIIb	-3.40±0.14	0.02
8	IIIc	-3.75±0.41	0.11
8	IIId	-4.38±0.64	0.05
8	IIIe	-3.90±0.59	0.04
8	IIIf	-4.70±0.80	0.04
8	IIIg	-4.28±0.52	0.06
8	IIIh	-4.37±0.57	0.07
9	adenosine	-4.88±0.17	0.02
9	IIa	-4.28±0.46	0.04
9	IIb	-4.78±0.29	0.03
9	IIc	-4.50±0.17	0.03
9	IId	-4.23±0.31	0.03
9	IIIa	-4.23±0.31	0.03
9	IIIb	-4.40±0.36	0.03
9	IIIc	-4.10±0.42	0.02
9	IIId	-4.33±0.50	0.04
9	IIIe	-4.15±0.49	0.02
9	IIIf	-4.05±0.13	0.04
9	IIIg	-3.77±0.15	0.03
9	IIIh	-4.48±0.17	0.04
10	adenosine	-5.11±0.21	0.06
10	caffeine	-4.30±0.00	0.08
10	IIa	-4.47±0.21	0.04

10	IIb	-4.65±0.47	0.03
10	IIc	-4.35±0.44	0.04
10	IId	-4.63±0.51	0.03
10	IIIa	-3.85±0.24	0.08
10	IIIb	-3.98±0.36	0.05
10	IIIc	-3.81±0.31	0.07
10	IIId	-4.00±0.35	0.08
10	IIIe	-3.97±0.21	0.06
10	IIIf	-4.20±0.18	0.08
10	IIIg	-4.09±0.47	0.08
10	IIIh	-4.33±0.30	0.04
11	adenosine	-5.92±0.58	0.03
11	caffeine	-4.85±0.26	0.33
11	IIa	-4.93±0.06	0.03
11	IId	-5.10±nan	0.01
11	IIIa	-4.50±0.28	0.02
11	IIIb	-4.90±nan	0.01
12	adenosine	-5.08±0.11	0.03
12	caffeine	-4.30±0.00	0.03
12	IIb	-4.40±0.28	0.02
12	IIc	-4.63±0.35	0.03
12	IId	-3.75±0.21	0.02
12	IIIa	-4.00±0.61	0.03
12	IIIb	-3.88±0.25	0.06
12	IIIc	-3.90±nan	0.01
12	IIId	-4.10±nan	0.01
12	IIIe	-3.55±0.44	0.04
12	IIIf	-4.58±0.57	0.04
12	IIIg	-4.10±0.26	0.03
12	IIIh	-5.00±nan	0.01
14	adenosine	-4.82±0.32	0.02
14	IIa	-4.05±0.49	0.02
14	IIb	-4.43±0.40	0.03
14	IIc	-4.33±0.43	0.04
14	IId	-4.47±0.06	0.03
14	IIIa	-4.10±0.44	0.03
14	IIIb	-4.20±0.45	0.04
14	IIIc	-3.85±0.33	0.06
14	IIId	-3.92±0.24	0.05
14	IIIe	-4.12±0.38	0.04
14	IIIf	-4.40±0.71	0.02
14	IIIg	-3.90±0.58	0.04
14	IIIh	-4.72±0.74	0.04
**15**	adenosine	-5.05±0.47	0.16

**15**	IIa	-4.60±0.48	0.46
**15**	IIb	-4.77±0.48	0.31
**15**	IIc	-4.81±0.45	0.3
**15**	IId	-4.79±0.53	0.28
**15**	IIIa	-3.93±0.37	0.33
**15**	IIIb	-4.33±0.43	0.33
**15**	IIIc	-4.17±0.51	0.3
**15**	IIId	-4.29±0.40	0.33
**15**	IIIe	-3.91±0.42	0.26
**15**	IIIf	-4.41±0.40	0.43
**15**	IIIg	-4.22±0.40	0.44
**15**	IIIh	-4.46±0.48	0.39
16	adenosine	-4.70±nan	0.01
16	IIa	-4.24±0.32	0.05
16	IIb	-4.39±0.32	0.1
16	IIc	-4.17±0.29	0.07
16	IId	-4.15±0.24	0.12
16	IIIa	-3.88±0.41	0.09
16	IIIb	-4.10±0.26	0.03
16	IIIc	-3.88±0.26	0.06
16	IIId	-3.90±nan	0.01
16	IIIe	-3.77±0.51	0.06
16	IIIg	-3.70±nan	0.01
16	IIIh	-3.90±0.10	0.03

Table S3. Affinities of the clusters for the A2AR receptor variant 5mzp

Cluster	Substance	Energy	Frequency
0	adenosine	-5.63±0.31	0.38
0	IIa	-4.70±0.33	0.09
0	IId	-5.15±0.07	0.02
0	IIIb	-4.65±0.35	0.03
0	IIIc	-4.40±nan	0.01
0	IIId	-4.70±nan	0.01
0	IIIe	-4.75±0.07	0.04
0	IIIh	-4.90±nan	0.01
1	adenosine	-4.92±0.23	0.06
1	IIa	-4.41±0.34	0.12
1	IIb	-4.55±0.35	0.03
1	IIc	-4.60±0.49	0.08
1	IId	-4.68±0.39	0.17
1	IIIa	-3.86±0.14	0.09
1	IIIb	-4.10±0.26	0.04
1	IIIc	-3.92±0.41	0.06
1	IIId	-4.08±0.15	0.06

1	IIIe	-4.23±0.60	0.06
1	IIIf	-4.10±0.00	0.03
1	IIIh	-4.27±0.15	0.04
2	adenosine	-6.40±0.14	0.03
2	caffeine	-4.64±0.13	0.33
2	IIa	-5.70±nan	0.01
2	IIIa	-5.00±0.14	0.03
2	IIIb	-4.70±0.20	0.04
2	IIIc	-5.20±nan	0.01
2	IIIe	-4.10±nan	0.02
2	IIIg	-4.90±nan	0.01
3	adenosine	-5.10±nan	0.01
3	IIa	-4.54±0.40	0.1
3	IIb	-4.78±0.25	0.07
3	IIc	-4.65±0.50	0.05
3	IId	-4.67±0.26	0.07
3	IIIa	-4.20±0.26	0.04
3	IIIb	-4.40±0.14	0.03
3	IIIc	-4.90±nan	0.01
3	IIId	-4.55±0.07	0.02
3	IIIe	-4.65±0.35	0.04
3	IIIf	-4.75±0.27	0.08
3	IIIg	-4.28±0.13	0.07
4	adenosine	-5.25±0.43	0.1
4	caffeine	-4.54±0.09	0.03
4	IIa	-4.10±0.44	0.04
4	IIb	-4.54±0.59	0.1
4	IIc	-4.52±0.42	0.21
4	IId	-4.35±0.44	0.05
4	IIIa	-3.79±0.38	0.09
4	IIIb	-4.03±0.29	0.1
4	IIIc	-4.17±0.57	0.09
4	IIId	-4.22±0.26	0.14
4	IIIe	-4.03±0.29	0.08
4	IIIf	-4.35±0.32	0.22
4	IIIg	-4.12±0.42	0.12
4	IIIh	-4.49±0.34	0.19
**5**	adenosine	-5.54±0.47	0.1
**5**	caffeine	-4.35±0.10	0.02
**5**	IIa	-4.74±0.34	0.32
**5**	IIb	-4.61±0.41	0.19
**5**	IIc	-4.71±0.30	0.23
**5**	IId	-5.01±0.34	0.2
**5**	IIIa	-4.38±0.53	0.19

**5**	IIIb	-4.38±0.40	0.31
**5**	IIIc	-4.41±0.37	0.27
**5**	IIId	-4.47±0.51	0.18
**5**	IIIe	-4.31±0.38	0.13
**5**	IIIf	-4.53±0.37	0.21
**5**	IIIg	-4.25±0.48	0.23
**5**	IIIh	-4.36±0.31	0.19
6	adenosine	-4.60±nan	0.01
6	IIa	-3.90±nan	0.01
6	IIb	-4.38±0.36	0.07
6	IIc	-4.16±0.29	0.07
6	IId	-4.23±0.33	0.07
6	IIIa	-4.05±0.49	0.03
6	IIIc	-3.80±0.08	0.05
6	IIId	-3.93±0.31	0.03
6	IIIe	-4.08±0.46	0.08
6	IIIf	-4.20±0.48	0.05
6	IIIg	-3.85±0.07	0.03
6	IIIh	-4.15±0.37	0.05
7	caffeine	-4.70±0.00	0.02
7	IIa	-4.69±0.47	0.1
7	IIb	-4.50±0.32	0.07
7	IIc	-4.63±0.46	0.04
7	IId	-4.70±0.44	0.03
7	IIIa	-4.10±0.32	0.08
7	IIIb	-4.26±0.39	0.14
7	IIIc	-3.94±0.57	0.06
7	IIId	-4.48±0.55	0.15
7	IIIe	-4.24±0.36	0.09
7	IIIf	-4.33±0.15	0.04
7	IIIg	-4.50±0.53	0.05
7	IIIh	-4.72±0.29	0.07
8	adenosine	-5.00±0.14	0.03
8	IIa	-4.30±nan	0.01
8	IIb	-4.68±0.44	0.16
8	IIc	-4.84±0.37	0.1
8	IId	-4.58±0.37	0.09
8	IIIa	-4.30±nan	0.01
8	IIIb	-3.50±nan	0.01
8	IIIc	-4.13±0.21	0.04
8	IIId	-4.85±0.92	0.02
8	IIIe	-3.95±0.07	0.04
8	IIIf	-4.28±0.28	0.08
8	IIIg	-4.08±0.24	0.07

8	IIIh	-4.30±0.00	0.03
10	adenosine	-6.80±nan	0.01
10	caffeine	-5.05±0.12	0.24
10	IIa	-4.95±0.21	0.03
10	IId	-6.60±nan	0.01
10	IIIa	-4.80±0.14	0.03
10	IIIb	-5.30±nan	0.01
10	IIIc	-5.40±nan	0.01
10	IIId	-5.60±nan	0.01
11	caffeine	-5.65±0.22	0.14
11	IIb	-5.80±nan	0.01
11	IIIa	-5.66±0.36	0.07
11	IIIc	-6.15±0.21	0.03
11	IIId	-6.80±nan	0.01
11	IIIe	-6.33±0.38	0.06
12	adenosine	-5.38±0.43	0.15
12	IIa	-4.35±0.17	0.06
12	IIb	-4.20±nan	0.01
12	IIc	-4.70±0.22	0.05
12	IId	-4.50±0.22	0.05
12	IIIa	-4.32±0.44	0.12
12	IIIb	-4.20±0.33	0.08
12	IIIc	-4.20±0.23	0.06
12	IIId	-4.63±0.28	0.07
12	IIIe	-4.36±0.33	0.09
12	IIIf	-4.02±0.21	0.08
12	IIIg	-4.14±0.37	0.11
12	IIIh	-4.37±0.24	0.1
13	adenosine	-5.50±nan	0.01
13	caffeine	-4.69±0.14	0.21
13	IIa	-5.00±0.14	0.03
13	IIb	-5.15±0.07	0.03
13	IIIg	-4.80±nan	0.01
14	adenosine	-4.70±nan	0.01
14	IIa	-3.55±0.21	0.03
14	IIb	-4.05±0.17	0.06
14	IIc	-4.03±0.34	0.05
14	IId	-4.10±0.42	0.02
14	IIIa	-4.03±0.42	0.04
14	IIIb	-3.90±nan	0.01
14	IIIc	-3.67±0.27	0.08
14	IIId	-3.90±0.28	0.05
14	IIIe	-3.88±0.23	0.09
14	IIIf	-3.90±0.71	0.03

14	IIIg	-3.72±0.28	0.07
15	adenosine	-4.70±nan	0.01
15	IIb	-4.00±0.28	0.03
15	IIc	-4.20±0.46	0.04
15	IId	-4.32±0.29	0.06
15	IIIa	-3.90±0.00	0.03
15	IIIb	-3.65±0.21	0.03
15	IIIc	-4.30±0.28	0.03
15	IIId	-4.02±0.19	0.06
15	IIIf	-4.32±0.51	0.05
15	IIIg	-3.72±0.13	0.07
15	IIIh	-4.20±0.40	0.04
16	adenosine	-4.72±0.21	0.05
16	IIb	-3.80±0.14	0.03
16	IIc	-4.28±0.28	0.05
16	IId	-4.20±0.22	0.05
16	IIIa	-3.70±0.00	0.03
16	IIIb	-4.15±0.21	0.06
16	IIIc	-3.55±0.64	0.03
16	IIId	-3.98±0.25	0.05
16	IIIe	-3.77±0.12	0.06
16	IIIf	-3.78±0.28	0.06
16	IIIg	-3.96±0.31	0.09
16	IIIh	-4.20±0.23	0.05
17	adenosine	-5.55±0.07	0.03
17	IIa	-4.00±0.42	0.03
17	IIb	-4.17±0.20	0.09
17	IIc	-4.50±nan	0.01
17	IId	-4.03±0.21	0.05
17	IIIa	-3.72±0.18	0.07
17	IIIb	-3.87±0.46	0.08
17	IIIc	-3.63±0.26	0.12
17	IIId	-3.91±0.24	0.09
17	IIIe	-3.78±0.13	0.08
17	IIIf	-4.10±0.41	0.06
17	IIIg	-4.03±0.50	0.05
17	IIIh	-4.20±0.23	0.16

Table S4. Affinities of the clusters for the APRT variant 6hgs

Cluster	Substance	Energy	Frequency
**0**	gmp	-6.07±0.45	0.18
**0**	IIa	-4.57±0.70	0.15
**0**	IIb	-4.91±0.81	0.19
**0**	IIc	-4.74±0.34	0.12

**0**	IId	-4.53±0.68	0.1
**0**	IMP	-7.43±0.55	0.35
**0**	IIIa	-4.77±0.57	0.23
**0**	IIIb	-4.41±0.36	0.09
**0**	IIIc	-4.65±0.55	0.06
**0**	IIId	-4.53±0.58	0.12
**0**	IIIe	-4.60±0.56	0.05
**0**	IIIf	-4.47±0.33	0.14
**0**	IIIg	-4.63±0.51	0.07
**0**	IIIh	-4.50±0.17	0.04
1	gmp	-5.55±0.21	0.02
1	IIa	-4.35±0.39	0.06
1	IIb	-4.42±0.45	0.06
1	IIc	-4.01±0.36	0.1
1	IId	-4.44±0.32	0.1
1	IMP	-6.35±0.07	0.02
1	IIIa	-4.11±0.33	0.13
1	IIIb	-4.40±0.14	0.03
1	IIIc	-4.14±0.31	0.12
1	IIId	-4.30±0.38	0.05
1	IIIe	-4.07±0.30	0.14
1	IIIf	-4.27±0.21	0.15
1	IIIg	-4.18±0.19	0.06
1	IIIh	-4.12±0.30	0.05
2	gmp	-5.85±0.07	0.02
2	IIa	-4.48±0.26	0.17
2	IIb	-4.54±0.50	0.13
2	IIc	-4.60±0.33	0.08
2	IId	-4.74±0.39	0.07
2	IMP	-6.25±0.07	0.02
2	IIIa	-4.26±0.47	0.12
2	IIIb	-4.46±0.31	0.16
2	IIIc	-4.25±0.68	0.06
2	IIId	-4.06±0.17	0.09
2	IIIe	-3.97±0.28	0.2
2	IIIf	-4.29±0.29	0.12
2	IIIg	-4.14±0.29	0.11
2	IIIh	-4.18±0.23	0.19
3	gmp	-6.10±0.12	0.11
3	IIa	-5.24±0.38	0.17
3	IIb	-5.17±0.40	0.04
3	IIc	-5.46±0.56	0.09
3	IId	-5.25±0.07	0.03
3	IMP	-7.04±0.33	0.17

3	IIIa	-5.30±0.28	0.03
3	IIIb	-6.50±nan	0.01
3	IIIc	-5.47±0.57	0.04
3	IIIf	-4.70±nan	0.01
3	IIIg	-6.00±nan	0.01
4	gmp	-5.77±0.32	0.1
4	IIa	-4.22±0.25	0.06
4	IIb	-4.24±0.49	0.23
4	IIc	-4.78±0.26	0.05
4	IId	-4.70±0.35	0.09
4	IMP	-6.88±0.13	0.05
4	IIIa	-3.93±0.46	0.08
4	IIIb	-4.40±0.40	0.09
4	IIIc	-4.15±0.19	0.09
4	IIId	-4.24±0.37	0.06
4	IIIe	-4.10±0.14	0.06
4	IIIf	-4.75±0.21	0.05
4	IIIg	-4.17±0.29	0.12
4	IIIh	-4.69±0.35	0.14
5	gmp	-5.70±0.21	0.17
5	IIa	-4.40±0.26	0.1
5	IIb	-4.63±0.51	0.11
5	IIc	-4.79±0.36	0.09
5	IId	-4.55±0.28	0.16
5	IMP	-6.82±0.34	0.11
5	IIIa	-3.97±0.42	0.08
5	IIIb	-4.37±0.28	0.18
5	IIIc	-3.92±0.25	0.12
5	IIId	-4.35±0.39	0.19
5	IIIe	-3.95±0.37	0.15
5	IIIf	-4.33±0.21	0.09
5	IIIg	-3.93±0.29	0.09
5	IIIh	-4.43±0.37	0.14
6	gmp	-5.91±0.27	0.13
6	IIa	-4.15±0.49	0.03
6	IIb	-4.50±0.69	0.04
6	IIc	-4.35±0.07	0.03
6	IMP	-6.75±0.07	0.02
6	IIIa	-4.30±0.57	0.03
6	IIIb	-4.20±0.35	0.04
6	IIIc	-4.07±0.51	0.09
6	IIId	-4.30±0.16	0.13
6	IIIe	-3.93±0.26	0.09
6	IIIf	-4.40±0.25	0.12

6	IIIg	-4.24±0.58	0.1
6	IIIh	-4.55±0.50	0.08
7	gmp	-5.70±0.14	0.02
7	IIa	-4.34±0.32	0.11
7	IIb	-4.38±0.48	0.06
7	IIc	-4.40±0.37	0.17
7	IId	-4.39±0.44	0.14
7	IMP	-6.30±nan	0.01
7	IIIa	-4.10±0.31	0.09
7	IIIb	-4.60±0.39	0.08
7	IIIc	-4.37±0.50	0.13
7	IIId	-4.20±0.17	0.06
7	IIIe	-3.97±0.26	0.07
7	IIIf	-4.23±0.42	0.04
7	IIIg	-4.08±0.39	0.12
7	IIIh	-4.42±0.24	0.07
8	gmp	-7.01±0.45	0.15
8	IIa	-4.90±0.45	0.08
8	IIb	-5.02±0.83	0.06
8	IId	-5.18±0.96	0.16
8	IMP	-7.34±0.83	0.12
8	IIIa	-4.82±0.45	0.17
8	IIIb	-4.91±0.66	0.2
8	IIIc	-4.64±0.61	0.14
8	IIId	-4.64±0.46	0.19
8	IIIe	-4.20±0.48	0.05
8	IIIf	-4.81±0.40	0.11
8	IIIg	-4.54±0.70	0.1
8	IIIh	-4.99±0.87	0.12
9	gmp	-5.60±nan	0.01
9	IIa	-4.70±nan	0.01
9	IIb	-4.70±0.14	0.03
9	IIc	-4.26±0.29	0.1
9	IId	-4.60±0.21	0.11
9	IMP	-6.35±0.07	0.02
9	IIIa	-3.80±0.35	0.04
9	IIIb	-4.15±0.10	0.05
9	IIIc	-4.38±0.27	0.07
9	IIId	-4.55±0.21	0.03
9	IIIe	-4.00±0.29	0.07
9	IIIf	-4.29±0.43	0.16
9	IIIg	-4.16±0.34	0.09
9	IIIh	-4.49±0.38	0.1
10	gmp	-5.64±0.25	0.08

10	IIa	-4.32±0.31	0.07
10	IIb	-4.15±0.40	0.05
10	IIc	-4.37±0.43	0.17
10	IId	-4.10±0.35	0.04
10	IMP	-6.54±0.16	0.09
10	IIIa	-4.20±0.00	0.03
10	IIIb	-4.20±0.18	0.05
10	IIIc	-4.23±0.27	0.09
10	IIId	-4.16±0.15	0.06
10	IIIe	-4.02±0.40	0.11
10	IIIg	-4.04±0.24	0.12
10	IIIh	-4.30±0.23	0.07

Table S5. Affinities of the clusters for the APRT variant 6hgr

Cluster	Substance	Energy	Frequency
0	gmp	-6.67±0.58	0.19
0	IIa	-4.79±0.41	0.09
0	IIb	-4.92±0.60	0.07
0	IIc	-4.80±0.67	0.21
0	IId	-5.33±0.45	0.07
0	IMP	-8.18±0.68	0.36
0	IIIa	-4.59±0.56	0.14
0	IIIb	-4.65±0.51	0.05
0	IIIc	-4.81±0.65	0.1
0	IIId	-4.65±0.39	0.07
0	IIIe	-5.17±0.79	0.07
0	IIIf	-4.83±0.68	0.05
0	IIIg	-4.30±0.26	0.03
0	IIIh	-5.03±0.81	0.05
1	gmp	-6.10±0.08	0.04
1	IIa	-4.30±0.14	0.02
1	IIb	-4.10±0.29	0.06
1	IIc	-4.25±0.32	0.15
1	IId	-4.33±0.62	0.05
1	IMP	-7.10±0.14	0.06
1	IIIa	-4.06±0.39	0.06
1	IIIb	-4.14±0.08	0.08
1	IIIc	-4.22±0.27	0.1
1	IIId	-4.12±0.13	0.06
1	IIIe	-4.02±0.24	0.14
1	IIIf	-4.35±0.43	0.1
1	IIIg	-4.20±0.22	0.05
1	IIIh	-4.59±0.41	0.09
2	gmp	-6.71±0.69	0.27

2	IIa	-5.00±0.44	0.15
2	IIb	-4.79±0.55	0.16
2	IIc	-4.25±0.24	0.05
2	IId	-4.72±0.77	0.14
2	IMP	-7.70±0.55	0.27
2	IIIa	-4.32±0.49	0.13
2	IIIb	-4.78±0.59	0.11
2	IIIc	-4.43±0.52	0.07
2	IIId	-4.79±0.42	0.1
2	IIIe	-4.27±0.31	0.05
2	IIIf	-4.62±0.62	0.15
2	IIIg	-4.65±0.56	0.2
2	IIIh	-4.78±0.58	0.13
3	gmp	-6.17±0.27	0.1
3	IIa	-4.60±0.53	0.12
3	IIb	-4.30±0.41	0.06
3	IIc	-4.53±0.23	0.11
3	IId	-4.17±0.68	0.04
3	IMP	-6.80±0.37	0.04
3	IIIa	-4.16±0.32	0.06
3	IIIb	-4.15±0.21	0.02
3	IIIc	-3.80±nan	0.01
3	IIId	-4.30±0.00	0.04
3	IIIe	-4.15±0.47	0.12
3	IIIf	-4.37±0.46	0.14
3	IIIg	-4.03±0.28	0.1
3	IIIh	-4.53±0.53	0.14
4	gmp	-5.85±0.35	0.02
4	IIb	-4.43±0.35	0.12
4	IIc	-4.38±0.33	0.06
4	IId	-4.43±0.24	0.08
4	IIIa	-4.30±0.26	0.05
4	IIIb	-4.45±0.21	0.13
4	IIIc	-4.07±0.26	0.09
4	IIId	-4.43±0.25	0.04
4	IIIe	-4.02±0.50	0.05
4	IIIf	-4.23±0.11	0.09
4	IIIg	-4.08±0.29	0.07
4	IIIh	-4.18±0.42	0.11
5	gmp	-6.20±0.24	0.04
5	IIa	-4.60±0.37	0.06
5	IIb	-4.40±0.14	0.02
5	IIc	-4.20±0.57	0.02
5	IId	-4.16±0.35	0.06

5	IIIa	-4.11±0.32	0.22
5	IIIb	-4.24±0.42	0.14
5	IIIc	-4.30±0.48	0.07
5	IIId	-4.28±0.25	0.06
5	IIIe	-4.10±0.18	0.07
5	IIIf	-4.47±0.50	0.05
5	IIIg	-4.00±0.14	0.02
5	IIIh	-4.46±0.67	0.06
**6**	gmp	-6.16±0.23	0.28
**6**	IIa	-5.14±0.55	0.31
**6**	IIb	-4.72±0.75	0.19
**6**	IIc	-5.07±0.60	0.23
**6**	IId	-5.41±0.63	0.27
**6**	IMP	-7.24±0.49	0.28
**6**	IIIa	-4.33±0.40	0.13
**6**	IIIb	-4.44±0.53	0.13
**6**	IIIc	-4.40±0.50	0.32
**6**	IIId	-4.35±0.41	0.23
**6**	IIIe	-4.26±0.40	0.19
**6**	IIIf	-4.37±0.23	0.09
**6**	IIIg	-4.48±0.60	0.22
**6**	IIIh	-4.58±0.62	0.15
7	gmp	-5.80±0.14	0.02
7	IIa	-4.63±0.26	0.08
7	IIb	-4.56±0.30	0.08
7	IIc	-4.38±0.41	0.06
7	IId	-4.49±0.35	0.13
7	IIIa	-4.39±0.46	0.09
7	IIIb	-4.10±0.33	0.11
7	IIIc	-4.32±0.27	0.07
7	IIId	-4.14±0.49	0.06
7	IIIe	-3.71±0.40	0.08
7	IIIf	-4.14±0.18	0.18
7	IIIg	-4.00±0.18	0.08
7	IIIh	-4.20±nan	0.01
8	gmp	-6.30±nan	0.01
8	IIa	-4.45±0.21	0.02
8	IIb	-4.50±nan	0.01
8	IIc	-4.60±nan	0.01
8	IId	-4.55±0.07	0.02
8	IIIa	-4.10±0.14	0.03
8	IIIb	-4.17±0.44	0.05
8	IIId	-4.28±0.40	0.17
8	IIIe	-4.10±0.26	0.12

8	IIIf	-4.03±0.29	0.05
8	IIIg	-4.28±0.23	0.09
8	IIIh	-4.39±0.34	0.15
9	gmp	-6.15±0.35	0.02
9	IIa	-4.68±0.51	0.07
9	IIb	-4.43±0.46	0.14
9	IIc	-4.18±0.33	0.06
9	IId	-3.95±0.50	0.05
9	IIIa	-4.10±0.28	0.05
9	IIIb	-4.38±0.42	0.12
9	IIIc	-4.20±0.30	0.1
9	IIId	-4.22±0.38	0.1
9	IIIe	-4.20±0.53	0.05
9	IIIf	-4.25±0.21	0.08
9	IIIg	-4.32±0.42	0.09
9	IIIh	-4.70±0.28	0.03

Table S6. Affinities of the clusters for the APRT variant 6hgp

Cluster	Substance	Energy	Frequency
**0**	gmp	-6.62±0.42	0.34
**0**	IIa	-4.80±0.50	0.14
**0**	IIb	-4.90±nan	0.01
**0**	IIc	-5.19±0.58	0.23
**0**	IId	-4.92±0.36	0.17
**0**	IMP	-7.73±0.72	0.13
**0**	IIIa	-4.64±0.79	0.14
**0**	IIIb	-4.26±0.49	0.07
**0**	IIIc	-4.25±0.30	0.14
**0**	IIId	-4.43±0.32	0.1
**0**	IIIe	-4.67±0.50	0.14
**0**	IIIf	-4.39±0.31	0.14
**0**	IIIg	-4.64±0.66	0.15
**0**	IIIh	-4.10±0.10	0.05
1	gmp	-5.73±0.24	0.11
1	IIa	-4.46±0.47	0.12
1	IIb	-4.29±0.43	0.23
1	IIc	-4.57±0.41	0.13
1	IId	-4.36±0.55	0.15
1	IMP	-7.12±0.54	0.06
1	IIIa	-4.00±0.24	0.18
1	IIIb	-4.24±0.43	0.1
1	IIIc	-4.46±0.41	0.12
1	IIId	-4.10±0.16	0.07
1	IIIe	-4.10±0.20	0.12

1	IIIf	-4.75±0.36	0.15
1	IIIg	-4.09±0.33	0.15
1	IIIh	-4.51±0.42	0.24
2	gmp	-6.73±0.24	0.1
2	IIa	-5.30±nan	0.01
2	IMP	-8.26±0.44	0.38
2	IIIh	-6.30±nan	0.02
3	gmp	-6.30±nan	0.02
3	IIa	-4.20±0.28	0.03
3	IIb	-4.17±0.24	0.11
3	IIc	-4.57±0.31	0.14
3	IId	-4.22±0.24	0.13
3	IMP	-6.82±0.55	0.15
3	IIIa	-4.08±0.22	0.13
3	IIIb	-4.28±0.45	0.12
3	IIIc	-4.25±0.46	0.21
3	IIId	-4.44±0.34	0.12
3	IIIe	-3.95±0.33	0.14
3	IIIf	-4.44±0.32	0.11
3	IIIg	-4.27±0.25	0.06
3	IIIh	-4.33±0.13	0.06
4	gmp	-6.10±nan	0.02
4	IIa	-4.22±0.30	0.06
4	IIb	-4.08±0.41	0.09
4	IIc	-3.95±0.21	0.03
4	IId	-4.82±0.33	0.05
4	IMP	-7.20±0.42	0.03
4	IIIa	-3.95±0.14	0.08
4	IIIb	-4.12±0.16	0.08
4	IIIc	-4.10±0.24	0.07
4	IIId	-4.25±0.37	0.12
4	IIIe	-4.00±0.21	0.09
4	IIIf	-4.58±0.23	0.14
4	IIIg	-4.58±0.54	0.06
4	IIIh	-4.40±0.39	0.08
5	gmp	-6.16±0.28	0.11
5	IIa	-4.70±0.42	0.03
5	IIb	-4.26±0.63	0.1
5	IIc	-4.47±0.46	0.04
5	IId	-4.59±0.12	0.09
5	IMP	-6.55±0.06	0.05
5	IIIa	-4.40±0.57	0.03
5	IIIb	-4.23±0.23	0.04
5	IIIc	-4.23±0.36	0.09

5	IIId	-4.26±0.30	0.07
5	IIIe	-3.92±0.39	0.06
5	IIIf	-4.48±0.26	0.08
5	IIIg	-4.30±0.63	0.09
5	IIIh	-4.51±0.37	0.13
6	gmp	-6.14±0.40	0.24
6	IIa	-5.06±0.49	0.33
6	IIb	-5.10±0.52	0.1
6	IIc	-5.13±0.64	0.1
6	IId	-5.27±0.60	0.16
6	IMP	-7.17±0.45	0.2
6	IIIa	-4.54±0.79	0.07
6	IIIb	-5.56±0.71	0.15
6	IIIc	-4.57±0.78	0.14
6	IIId	-4.31±0.22	0.12
6	IIIe	-4.57±0.45	0.1
6	IIIf	-4.37±0.40	0.08
6	IIIg	-4.80±0.48	0.09
6	IIIh	-5.12±0.82	0.15
7	gmp	-6.00±nan	0.02
7	IIa	-4.48±0.50	0.09
7	IIb	-4.00±0.52	0.09
7	IIc	-4.35±0.56	0.06
7	IId	-4.31±0.66	0.11
7	IIIa	-4.17±0.40	0.11
7	IIIb	-4.57±0.59	0.14
7	IIIc	-4.07±0.33	0.08
7	IIId	-4.40±0.31	0.1
7	IIIe	-4.30±0.61	0.06
7	IIIf	-4.20±0.17	0.09
7	IIIg	-3.84±0.22	0.07
7	IIIh	-4.43±0.29	0.06
9	gmp	-5.50±0.46	0.05
9	IIa	-4.59±0.35	0.16
9	IIb	-4.46±0.43	0.2
9	IIc	-4.21±0.49	0.13
9	IId	-4.31±0.43	0.09
9	IIIa	-3.93±0.14	0.1
9	IIIb	-4.63±0.41	0.14
9	IIIc	-3.90±0.16	0.07
9	IIId	-4.33±0.42	0.04
9	IIIe	-3.83±0.25	0.1
9	IIIf	-4.45±0.35	0.11
9	IIIg	-4.07±0.40	0.16

9	IIIh	-4.60±0.36	0.05

Table S7. Affinities of the clusters for the CDK2 variant 5fp5

Cluster	Substance	Energy	Frequency
**0**	IIa	-4.48±0.51	0.18
**0**	IIb	-4.48±0.38	0.14
**0**	IIc	-4.83±0.44	0.19
**0**	IId	-4.41±0.38	0.12
**0**	inh	-4.97±0.44	0.18
**0**	IIIa	-4.13±0.38	0.3
**0**	IIIb	-4.39±0.49	0.27
**0**	IIIc	-3.93±0.35	0.14
**0**	IIId	-4.30±0.61	0.17
**0**	IIIe	-3.98±0.43	0.14
**0**	IIIf	-4.32±0.46	0.19
**0**	IIIg	-4.11±0.41	0.18
**0**	IIIh	-4.54±0.31	0.17
1	IIa	-4.28±0.79	0.07
1	IIb	-4.30±0.14	0.03
1	IIc	-4.20±nan	0.02
1	IId	-4.22±0.81	0.07
1	inh	-4.70±0.20	0.05
1	IIIb	-4.24±0.25	0.08
1	IIIc	-3.85±0.19	0.06
1	IIId	-4.10±0.00	0.03
1	IIIe	-4.20±0.67	0.07
1	IIIf	-4.67±0.44	0.14
1	IIIh	-4.30±0.14	0.03
2	IIa	-4.54±0.38	0.12
2	IIb	-4.30±0.44	0.05
2	IIc	-4.90±0.55	0.07
2	IId	-4.90±0.55	0.07
2	inh	-4.70±0.17	0.05
2	IIIa	-3.90±0.12	0.07
2	IIIb	-4.03±0.12	0.05
2	IIIc	-3.90±0.15	0.09
2	IIId	-4.30±0.39	0.08
2	IIIe	-3.78±0.08	0.08
2	IIIf	-3.97±0.31	0.05
2	IIIg	-4.35±0.21	0.04
2	IIIh	-4.05±0.07	0.03
4	IIa	-3.85±0.07	0.03
4	IIb	-3.50±nan	0.02
4	IIc	-4.53±0.15	0.05

4	IId	-4.12±0.26	0.07
4	inh	-4.50±nan	0.02
4	IIIa	-3.70±0.42	0.03
4	IIIc	-4.00±0.32	0.08
4	IIId	-4.25±0.39	0.06
4	IIIe	-3.87±0.23	0.05
4	IIIf	-3.82±0.21	0.07
4	IIIg	-3.70±0.14	0.04
4	IIIh	-3.93±0.23	0.05
5	IIa	-4.20±0.24	0.06
5	IIb	-4.58±0.22	0.06
5	IIc	-4.65±0.35	0.03
5	IId	-4.13±0.29	0.05
5	inh	-4.80±0.17	0.09
5	IIIa	-3.58±0.10	0.07
5	IIIb	-4.02±0.43	0.07
5	IIIc	-3.88±0.30	0.08
5	IIId	-4.27±0.46	0.14
5	IIIe	-3.77±0.38	0.05
5	IIIf	-4.03±0.45	0.05
5	IIIg	-4.00±0.44	0.12
5	IIIh	-4.23±0.32	0.05
6	IIa	-4.30±nan	0.01
6	IIb	-4.02±0.22	0.06
6	IIc	-3.74±0.54	0.09
6	IId	-4.30±0.17	0.05
6	inh	-5.04±0.42	0.08
6	IIIb	-4.23±0.39	0.1
6	IIIc	-4.23±0.59	0.05
6	IIId	-4.28±0.29	0.09
6	IIIe	-4.24±0.40	0.08
6	IIIg	-3.82±0.16	0.09
6	IIIh	-4.20±0.22	0.07
7	IIb	-4.97±0.37	0.14
7	IIc	-4.99±0.58	0.12
7	IId	-4.70±nan	0.02
7	inh	-4.80±0.28	0.03
7	IIIa	-4.00±0.28	0.03
7	IIIb	-3.90±nan	0.02
7	IIIc	-4.30±nan	0.02
7	IIId	-5.50±0.14	0.03
7	IIIe	-3.75±0.21	0.03
7	IIIf	-3.97±0.17	0.07
7	IIIh	-5.80±nan	0.02

8	IIa	-4.35±0.21	0.03
8	IIb	-4.00±0.14	0.03
8	IIc	-3.70±nan	0.02
8	IId	-4.58±0.22	0.07
8	inh	-4.75±0.31	0.06
8	IIIb	-4.00±nan	0.02
8	IIIc	-4.20±nan	0.02
8	IIId	-4.17±0.22	0.06
8	IIIe	-4.10±nan	0.02
8	IIIf	-4.38±0.31	0.09
8	IIIg	-3.67±0.13	0.07
8	IIIh	-4.29±0.25	0.12
9	IIa	-4.33±0.05	0.06
9	IIb	-4.23±0.18	0.11
9	IIc	-4.30±0.37	0.07
9	IId	-4.46±0.38	0.08
9	inh	-4.98±0.19	0.08
9	IIIa	-3.90±0.00	0.03
9	IIIb	-4.20±nan	0.02
9	IIIc	-3.74±0.26	0.08
9	IIId	-3.90±nan	0.02
9	IIIe	-3.75±0.21	0.03
9	IIIf	-3.90±0.00	0.05
9	IIIg	-3.65±0.16	0.11
9	IIIh	-4.20±0.26	0.05
10	IIa	-5.85±0.07	0.03
10	IIc	-5.50±0.42	0.03
10	IId	-6.20±nan	0.02
10	inh	-6.35±0.40	0.12
10	IIIa	-5.10±0.71	0.03
10	IIIb	-5.60±1.41	0.03
10	IIIh	-6.35±0.21	0.03
11	IIb	-4.50±0.35	0.08
11	IIc	-4.27±0.67	0.05
11	IId	-4.57±0.45	0.05
11	inh	-4.81±0.23	0.11
11	IIIa	-3.62±0.26	0.07
11	IIIb	-4.13±0.31	0.05
11	IIIc	-3.99±0.33	0.15
11	IIId	-4.04±0.22	0.08
11	IIIe	-4.06±0.30	0.14
11	IIIf	-4.10±0.29	0.09
11	IIIg	-3.90±0.08	0.07
11	IIIh	-4.37±0.21	0.05

12	IIa	-4.16±0.39	0.07
12	IIb	-4.10±nan	0.02
12	IIc	-5.25±0.07	0.03
12	IId	-4.40±0.30	0.05
12	inh	-5.10±0.56	0.05
12	IIIa	-4.07±0.21	0.05
12	IIIb	-4.55±0.57	0.07
12	IIIc	-4.42±0.55	0.08
12	IIId	-3.90±nan	0.02
12	IIIe	-3.83±0.38	0.05
12	IIIf	-4.60±nan	0.02
12	IIIg	-4.05±0.25	0.07
12	IIIh	-4.42±0.22	0.07
13	IIa	-4.28±0.58	0.17
13	IIb	-4.00±0.37	0.09
13	IIc	-4.06±0.69	0.09
13	IId	-4.42±0.57	0.1
13	inh	-4.87±0.25	0.05
13	IIIa	-4.12±0.40	0.07
13	IIIb	-4.43±0.15	0.05
13	IIIc	-4.08±0.47	0.06
13	IIId	-3.97±0.25	0.05
13	IIIe	-3.92±0.26	0.1
13	IIIf	-4.50±0.42	0.03
13	IIIg	-3.54±0.11	0.09
13	IIIh	-4.57±0.59	0.1
14	IIa	-4.67±0.60	0.17
14	IIb	-4.42±0.25	0.2
14	IIc	-4.28±0.35	0.1
14	IId	-4.47±0.33	0.2
14	inh	-5.00±0.47	0.06
14	IIIa	-3.92±0.42	0.2
14	IIIb	-4.41±0.57	0.12
14	IIIc	-4.45±0.26	0.09
14	IIId	-4.19±0.42	0.12
14	IIIe	-3.91±0.44	0.14
14	IIIf	-3.88±0.26	0.09
14	IIIh	-4.27±0.38	0.05

Table S8. Affinities of the clusters for the CDK2 variant 2jgz

Cluster	Substance	Energy	Frequency
**0**	IIa	-4.87±0.32	0.16
**0**	IIb	-5.09±0.31	0.2
**0**	IIc	-5.21±0.45	0.24
**0**	IId	-5.14±0.47	0.2
**0**	inh	-5.68±0.33	0.22
**0**	IIIa	-4.89±0.66	0.12
**0**	IIIb	-4.88±0.44	0.17
**0**	IIIc	-4.51±0.42	0.1
**0**	IIId	-4.86±0.52	0.22
**0**	IIIe	-4.27±0.42	0.12
**0**	IIIf	-4.68±0.48	0.09
**0**	IIIg	-4.59±0.59	0.16
**0**	IIIh	-4.91±0.54	0.15
1	IIa	-4.80±nan	0.01
1	IIb	-4.70±nan	0.01
1	IIc	-4.80±0.14	0.01
1	inh	-5.66±0.13	0.02
1	IIIa	-4.70±0.22	0.02
1	IIIb	-5.00±0.41	0.03
1	IIIc	-4.57±0.23	0.02
1	IIId	-5.20±nan	0.01
1	IIIe	-4.60±nan	0.01
1	IIIf	-4.73±0.21	0.02
1	IIIg	-4.85±0.07	0.01
1	IIIh	-4.73±0.42	0.02
2	IIa	-4.45±0.37	0.02
2	IIb	-4.85±0.28	0.07
2	IIc	-4.82±0.46	0.06
2	IId	-4.68±0.27	0.04
2	inh	-5.25±0.27	0.03
2	IIIa	-4.30±0.09	0.03
2	IIIb	-4.86±0.35	0.04
2	IIIc	-4.36±0.29	0.05
2	IIId	-4.67±0.34	0.08
2	IIIe	-4.27±0.34	0.06
2	IIIf	-4.58±0.46	0.08
2	IIIg	-4.34±0.27	0.05
2	IIIh	-4.77±0.28	0.12
3	IIa	-4.88±0.42	0.07
3	IIb	-4.90±0.24	0.03
3	IIc	-4.85±0.29	0.05
3	IId	-4.85±0.56	0.08

3	inh	-5.44±0.27	0.08
3	IIIa	-4.68±0.56	0.07
3	IIIb	-4.82±0.25	0.05
3	IIIc	-4.70±0.46	0.12
3	IIId	-5.14±0.58	0.03
3	IIIe	-4.52±0.40	0.03
3	IIIf	-4.92±0.31	0.04
3	IIIg	-4.74±0.62	0.06
3	IIIh	-5.01±0.43	0.08
7	IIa	-4.66±0.38	0.11
7	IIb	-4.78±0.32	0.1
7	IIc	-4.54±0.33	0.07
7	IId	-4.85±0.43	0.06
7	inh	-5.39±0.32	0.07
7	IIIa	-4.19±0.36	0.09
7	IIIb	-4.53±0.40	0.08
7	IIIc	-4.37±0.38	0.12
7	IIId	-4.98±0.37	0.06
7	IIIe	-4.28±0.34	0.07
7	IIIf	-4.53±0.37	0.07
7	IIIg	-4.47±0.17	0.02
7	IIIh	-4.73±0.40	0.08
8	IIb	-5.06±0.17	0.03
8	IIc	-5.23±0.30	0.05
8	IId	-4.50±0.26	0.04
8	inh	-5.35±0.21	0.01
8	IIIa	-4.30±0.20	0.02
8	IIIb	-4.20±0.17	0.03
8	IIIc	-4.57±0.55	0.02
8	IIId	-4.43±0.12	0.02
8	IIIe	-4.35±0.40	0.04
8	IIIf	-4.36±0.55	0.03
8	IIIg	-4.37±0.49	0.02
8	IIIh	-4.60±0.44	0.02
9	IIa	-4.62±0.33	0.03
9	IIb	-4.73±0.27	0.05
9	IIc	-4.82±0.33	0.05
9	IId	-4.26±0.19	0.04
9	inh	-5.57±0.06	0.01
9	IIIa	-4.17±0.43	0.03
9	IIIb	-4.46±0.47	0.04
9	IIIc	-4.07±0.35	0.04
9	IIId	-4.67±0.22	0.02
9	IIIe	-4.59±0.68	0.04

9	IIIf	-4.60±0.34	0.03
9	IIIg	-4.20±0.00	0.01
9	IIIh	-4.40±nan	0.01
12	IIa	-4.75±0.52	0.03
12	IIb	-4.90±0.59	0.03
12	IIc	-5.01±0.39	0.05
12	IId	-5.42±0.43	0.06
12	inh	-5.58±0.24	0.09
12	IIIa	-4.48±0.42	0.07
12	IIIb	-4.84±0.18	0.04
12	IIIc	-4.46±0.28	0.03
12	IIId	-4.80±0.37	0.02
12	IIIe	-4.74±0.57	0.09
12	IIIf	-5.13±0.21	0.02
12	IIIg	-5.13±0.74	0.02
12	IIIh	-5.19±0.38	0.04
13	IIa	-4.70±0.37	0.13
13	IIb	-4.83±0.33	0.11
13	IIc	-4.76±0.20	0.07
13	IId	-4.63±0.49	0.13
13	inh	-5.38±0.18	0.11
13	IIIa	-4.44±0.45	0.07
13	IIIb	-4.58±0.40	0.09
13	IIIc	-4.22±0.38	0.1
13	IIId	-4.50±0.28	0.06
13	IIIe	-4.48±0.40	0.11
13	IIIf	-4.62±0.30	0.12
13	IIIg	-4.40±0.48	0.12
13	IIIh	-4.80±0.26	0.11
14	IIa	-4.63±0.35	0.06
14	IIb	-4.78±0.41	0.02
14	IIc	-4.93±0.42	0.02
14	IId	-4.58±0.31	0.03
14	inh	-5.51±0.25	0.14
14	IIIa	-4.66±0.43	0.11
14	IIIb	-4.99±0.49	0.09
14	IIIc	-4.33±0.31	0.04
14	IIId	-5.09±0.51	0.06
14	IIIe	-4.82±0.34	0.06
14	IIIf	-4.93±0.67	0.06
14	IIIg	-4.76±0.27	0.07
14	IIIh	-5.17±0.37	0.03
15	IIa	-4.45±0.07	0.01
15	IIb	-4.70±nan	0.01

15	IIc	-4.68±0.23	0.04
15	IId	-4.50±nan	0.01
15	inh	-5.34±0.20	0.06
15	IIIa	-4.14±0.34	0.03
15	IIIb	-4.28±0.22	0.03
15	IIIc	-4.26±0.16	0.04
15	IIId	-4.68±0.34	0.07
15	IIIe	-4.58±0.36	0.03
15	IIIf	-4.40±0.27	0.03
15	IIIg	-4.33±0.65	0.02
15	IIIh	-4.67±0.80	0.02
16	IIa	-4.50±nan	0.01
16	IIb	-4.30±0.55	0.02
16	IIc	-4.54±0.30	0.03
16	IId	-4.39±0.20	0.04
16	inh	-4.97±0.06	0.01
16	IIIa	-4.22±0.32	0.05
16	IIIb	-4.00±nan	0.01
16	IIIc	-4.02±0.25	0.03
16	IIId	-4.53±0.06	0.02
16	IIIe	-4.50±0.28	0.01
16	IIIf	-4.42±0.24	0.06
16	IIIg	-4.05±0.44	0.02
16	IIIh	-4.30±0.23	0.03
17	IIa	-4.60±0.14	0.01
17	IIb	-4.77±0.31	0.02
17	IIc	-4.52±0.33	0.03
17	IId	-4.64±0.29	0.03
17	inh	-5.17±0.12	0.01
17	IIIa	-3.86±0.37	0.05
17	IIIb	-4.36±0.33	0.06
17	IIIc	-4.05±0.31	0.02
17	IIId	-4.44±0.21	0.03
17	IIIe	-4.26±0.36	0.03
17	IIIf	-4.60±0.20	0.03
17	IIIg	-4.12±0.49	0.04
17	IIIh	-4.68±0.19	0.03
18	IIa	-4.45±0.39	0.06
18	IIb	-4.95±0.52	0.06
18	IIc	-4.81±0.49	0.04
18	IId	-4.67±0.56	0.04
18	inh	-5.45±0.16	0.06
18	IIIa	-4.37±0.47	0.06
18	IIIb	-4.47±0.23	0.07

18	IIIc	-4.50±0.30	0.05
18	IIId	-4.73±0.31	0.11
18	IIIe	-4.42±0.36	0.09
18	IIIf	-4.53±0.32	0.07
18	IIIg	-4.54±0.39	0.14
18	IIIh	-4.84±0.41	0.08
19	IIa	-4.76±0.34	0.04
19	IIb	-4.60±nan	0.01
19	IIc	-5.30±nan	0.01
19	inh	-5.57±0.18	0.03
19	IIIb	-4.53±0.35	0.02
19	IIIc	-4.30±nan	0.01
19	IIId	-4.67±0.60	0.02
19	IIIe	-4.10±0.14	0.01
19	IIIf	-4.57±0.45	0.02
19	IIIg	-3.95±0.07	0.01
19	IIIh	-4.50±nan	0.01
20	IIa	-4.78±0.31	0.07
20	IIb	-4.57±0.45	0.02
20	IIc	-4.96±0.29	0.04
20	IId	-4.85±0.07	0.01
20	inh	-5.20±nan	0
20	IIIb	-4.53±0.68	0.04
20	IIIc	-4.28±0.24	0.03
20	IIId	-4.50±0.28	0.01
20	IIIe	-4.57±0.67	0.02
20	IIIf	-4.35±0.21	0.01
20	IIIg	-4.63±0.06	0.02
20	IIIh	-4.97±0.29	0.02
22	IIa	-4.92±0.26	0.02
22	IIb	-4.78±0.58	0.03
22	IId	-4.83±0.21	0.02
22	inh	-5.44±0.23	0.02
22	IIIa	-4.27±0.44	0.05
22	IIIb	-4.70±0.10	0.02
22	IIIc	-4.93±0.41	0.03
22	IIId	-5.16±0.55	0.03
22	IIIe	-4.53±0.59	0.05
22	IIIf	-4.36±0.22	0.05
22	IIIg	-4.56±0.49	0.06
22	IIIh	-4.72±0.31	0.03
23	IIa	-4.86±0.42	0.06
23	IIb	-4.68±0.75	0.03
23	IIc	-4.40±nan	0.01

23	IId	-4.53±0.34	0.03
23	inh	-5.40±nan	0
23	IIIa	-4.31±0.52	0.04
23	IIIb	-4.13±0.12	0.02
23	IIIc	-4.30±0.10	0.02
23	IIId	-4.07±0.21	0.04
23	IIIe	-4.05±0.07	0.01
23	IIIf	-4.30±0.20	0.02
23	IIIg	-4.43±0.31	0.02
23	IIIh	-4.85±0.07	0.01
25	IIa	-4.70±nan	0.01
25	IIb	-5.20±nan	0.01
25	IIc	-5.00±nan	0.01
25	inh	-5.50±nan	0
25	IIIb	-4.90±nan	0.01
25	IIIe	-4.90±0.99	0.01
